# Supplementary material for: Miniscule differences between sex chromosomes in the giant genome of a salamander
Source: Sci Rep. 2018 Dec 14;8:17882. doi: 10.1038/s41598-018-36209-2 (PMC6294749; doi:10.1038/s41598-018-36209-2)
Supplement: Supplementary file 1 — Supplementary Information [file 41598_2018_36209_MOESM1_ESM.pdf]

Miniscule differences between sex chromosomes in the giant genome of a salamander

Melissa C. Keinath[1,2*], Nataliya Timoshevskaya[1], Vladimir A. Timoshevskiy[1], S. Randal Voss[3], Jeramiah J. Smith[1*]

[1] - Department of Biology, University of Kentucky, Lexington, KY, USA.
[2] - Department of Embryology, Carnegie Institution for Science, Baltimore, MD, USA.
[3] - Department of Neuroscience, Spinal Cord & Brain Injury Research Center (SCoBIRC), & Ambystoma Genetic Stock Center, University of Kentucky, Lexington, KY, USA.
* - Communicating Authors

SUPPLEMENTARY FIGURES

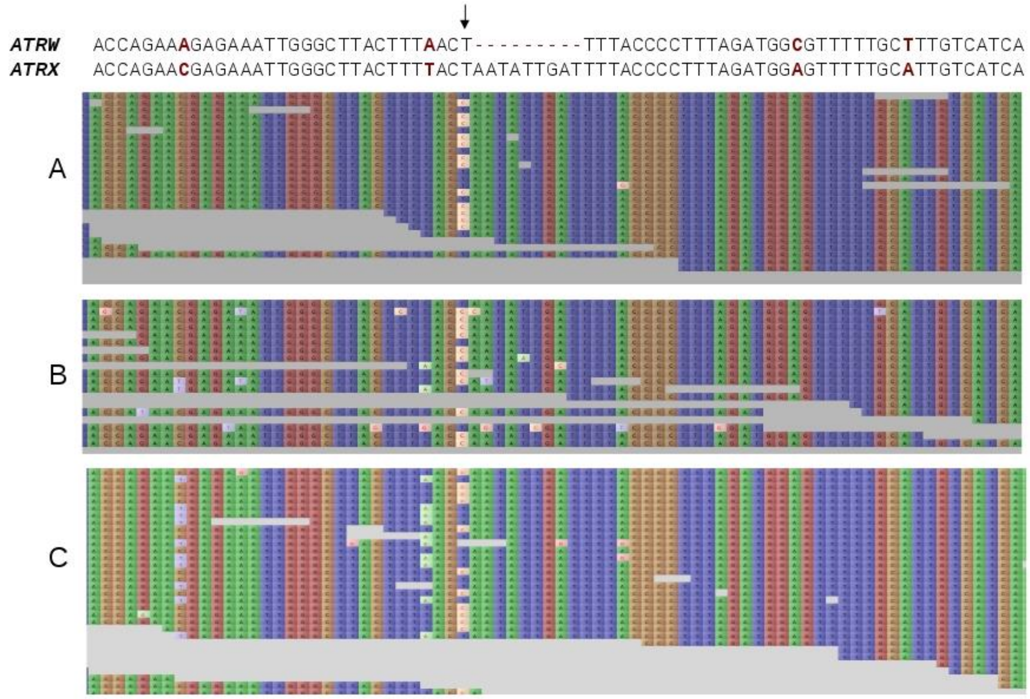

Supplementary Figure 1. Two alleles detected for *ATRX* in axolotl*.*

Two alleles can be observed in exon 9 of ATRX within the region homologous to the validated sex-specific DNA fragment of *ATRW* (SuperContig_990642). Alignment of short reads from genome sequencing of **A)** an *A. mexicanum* female, **B)** 22 female individuals, **C)** and 26 males to positions 6590-6665 of SuperContig_546209 (the contig containing exon 9 of the ATRX gene in the female genome assembly). The position marked with an arrow corresponds to position 1018 of SuperContig_990642 and position 6620 of SuperContig_546209. Visualization of the alignment produced using Tablet 1.14 [92].

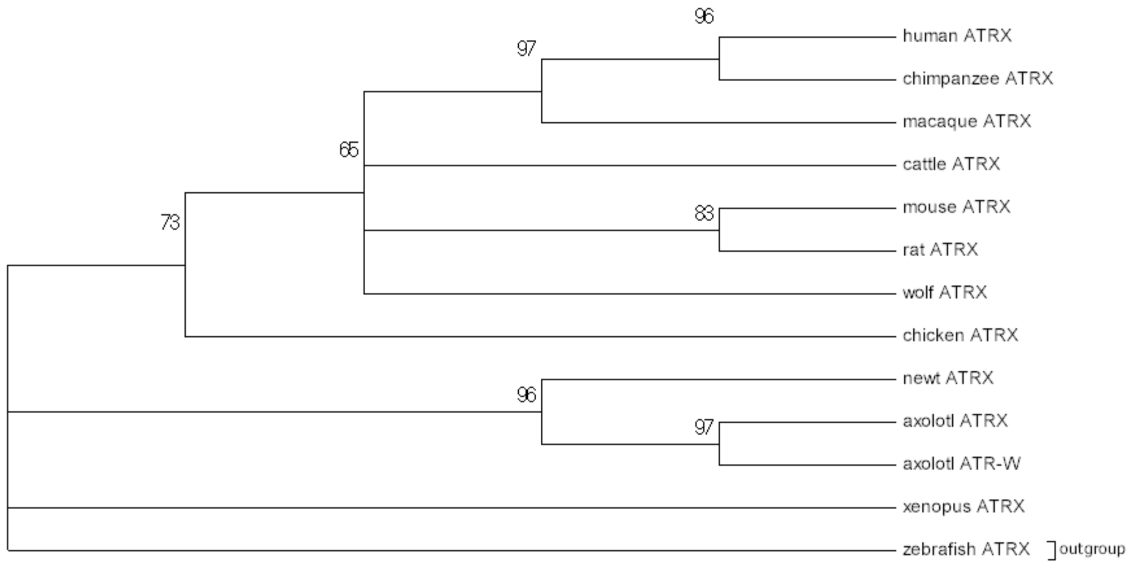

Supplementary Figure 2. Neighbor-Joining tree with bootstraps for vertebrate *ATRX.*

The evolutionary history was inferred using the Neighbor-Joining method [87]. The bootstrap consensus tree inferred from 10000 replicates is taken to represent the evolutionary history of the taxa analyzed [88]. Branches corresponding to partitions reproduced in less than 50% bootstrap replicates are collapsed. The percentage of replicate trees in which the associated taxa clustered together in the bootstrap test (10000 replicates) are shown next to the branches [88]. The evolutionary distances were computed using the Maximum Composite Likelihood method [89] and are in the units of the number of base substitutions per site. The analysis involved 13 nucleotide sequences. Codon positions included were 1st+2nd+3rd+Noncoding. All positions containing gaps and missing data were eliminated. There was a total of 251 positions in the final dataset. Evolutionary analyses were conducted in MEGA7 [84].

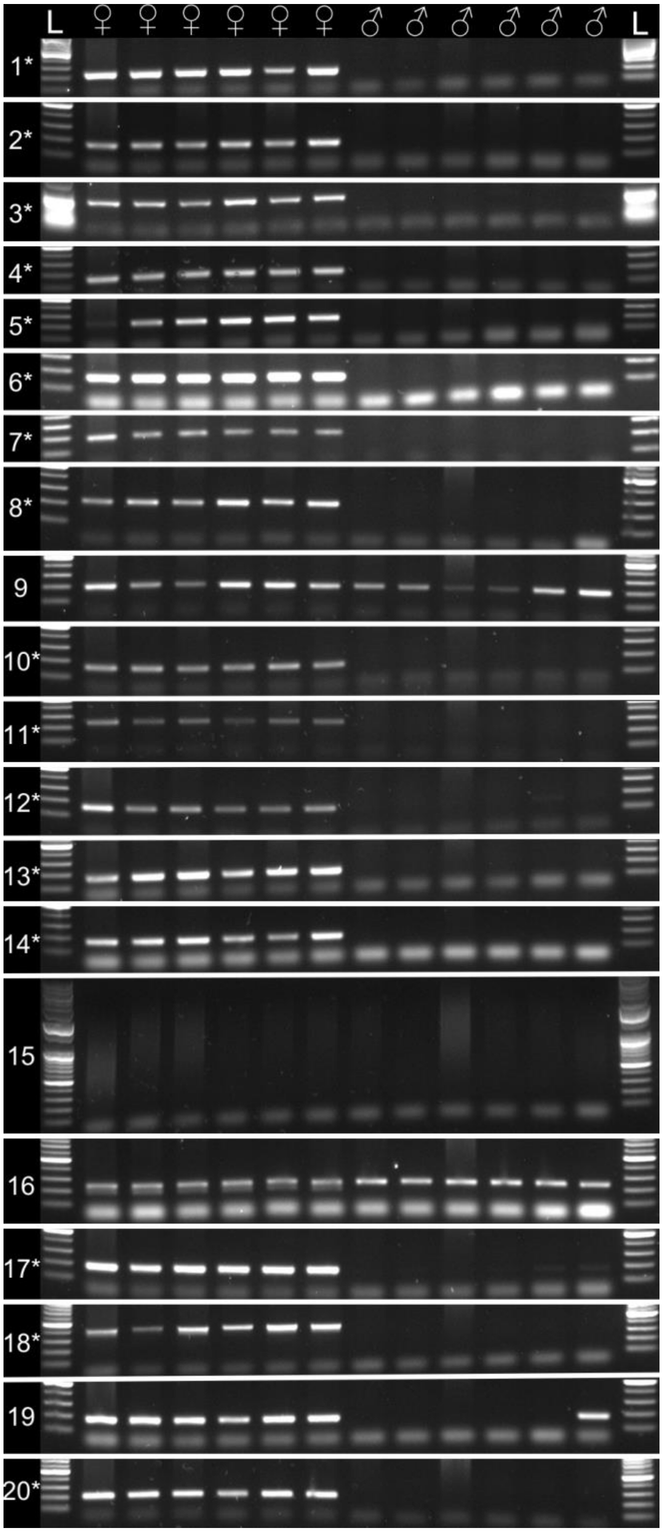

Supplementary Figure 3. PCR validation of sex-specific fragments.

Sample gel electrophoresis images of PCR for scaffolds determined to be sex-specific based on computational analyses. PCRs were tested on six females and six males, and

the associated lanes are denoted with ♀ and ♂, respectively. The first and last lanes are labeled with “L” to denote 100 bp ladder. Numerical labels correspond to primer information provided in Supplemental Table 3 along with the results of all PCRs tested. The images were cropped from full gel images (available in Supplemental Figure 4) to show presence/absence of fragments across individuals from multiple gel images and are separated by white lines. Those that show presence in females only are denoted with an asterisk and considered sex-specific. Also represented in this subset is an example of no amplification across any individuals (#15), amplification across all individuals (#9 and #16), and amplification across all females and 1/6 males (#19).

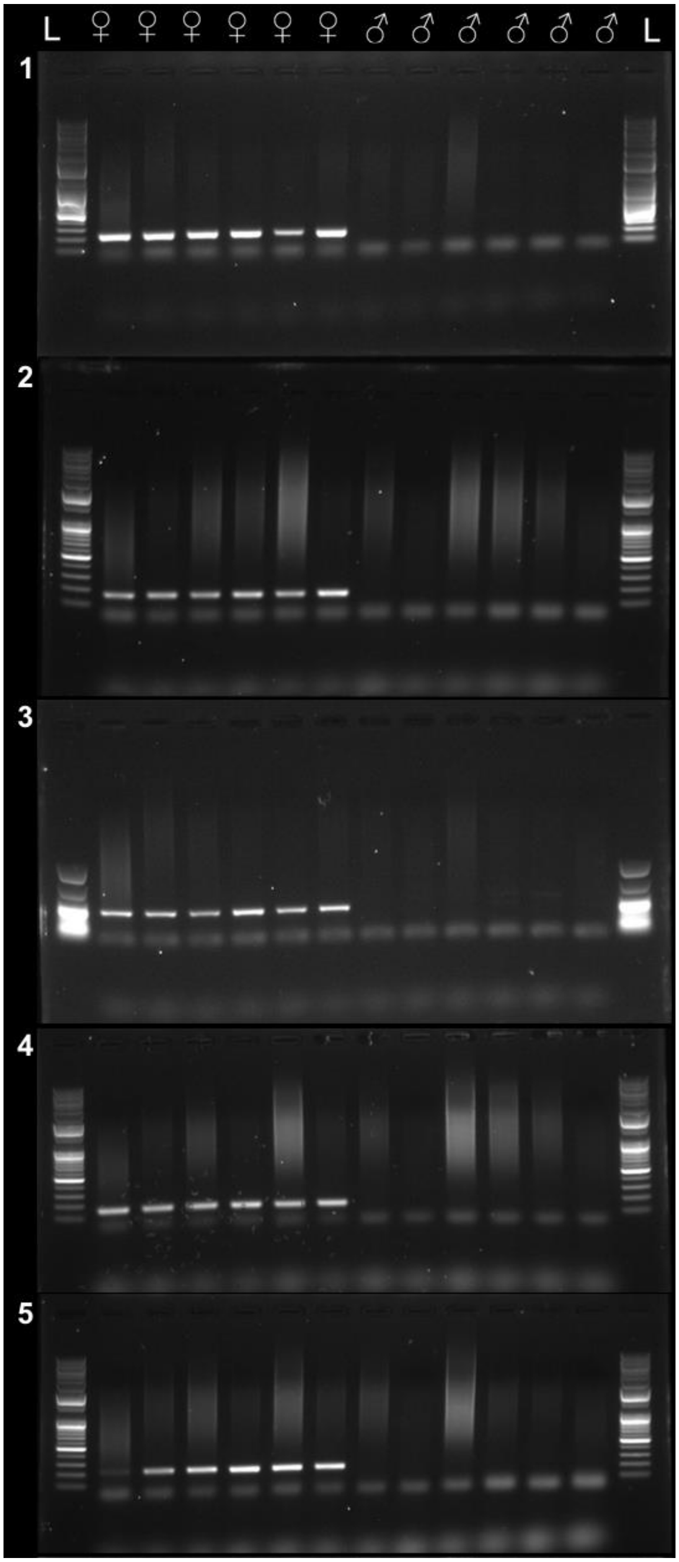
L ♀ ♀ ♀ ♀ ♀ ♀ ♂ ♂ ♂ ♂ ♂ ♂ L
1
2
3
4
5

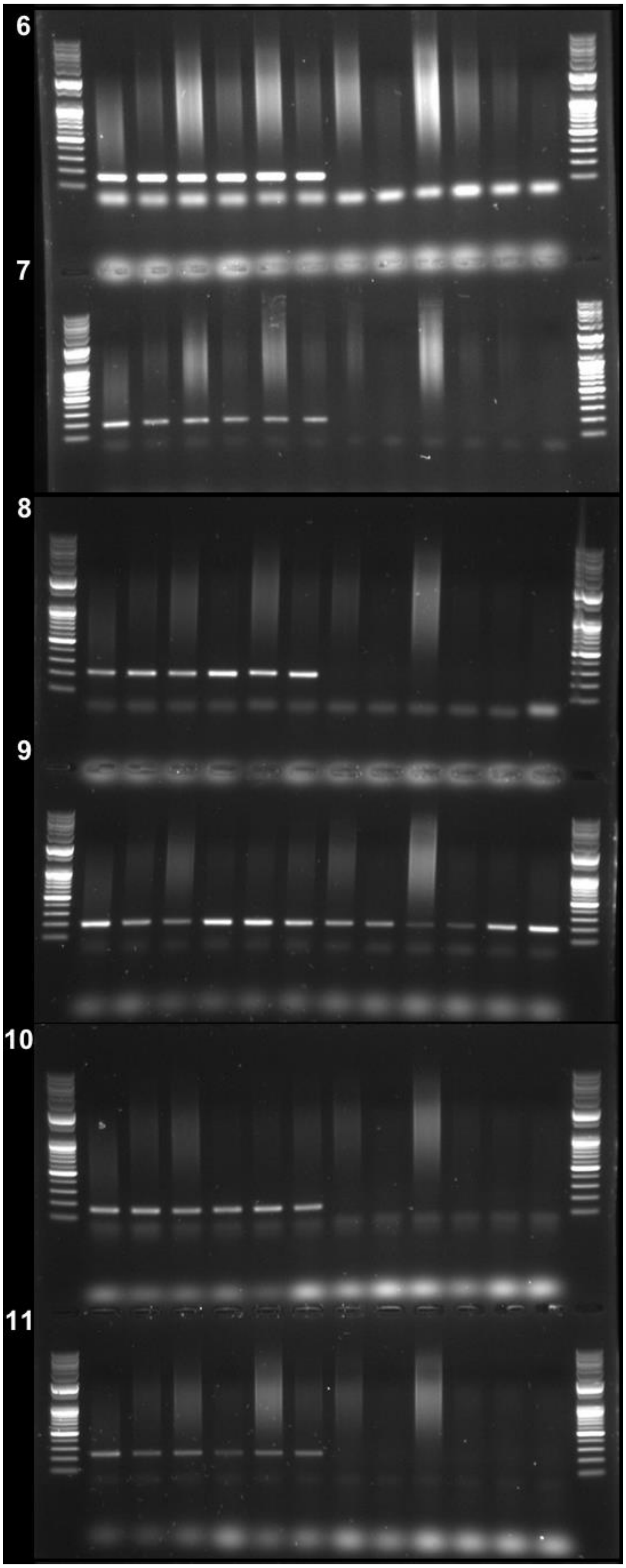
6
7
8
9
10
11

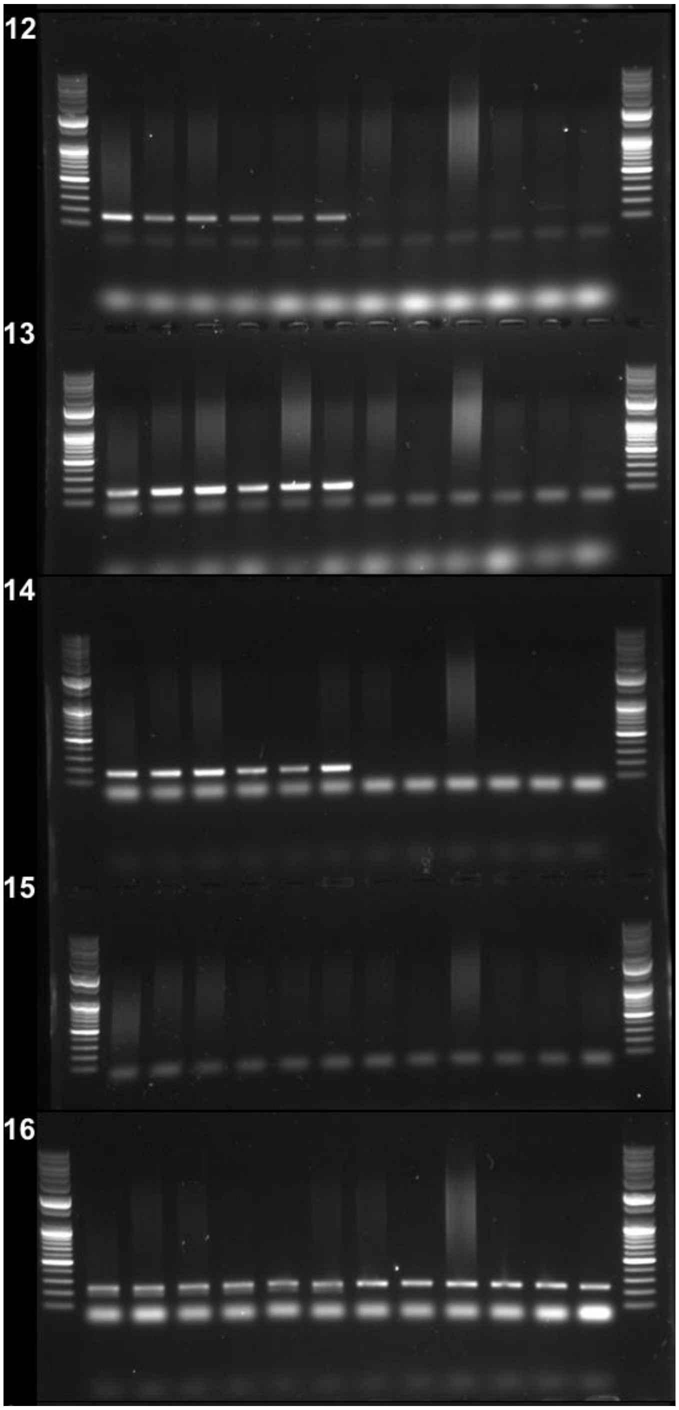
12
13
14
15
16

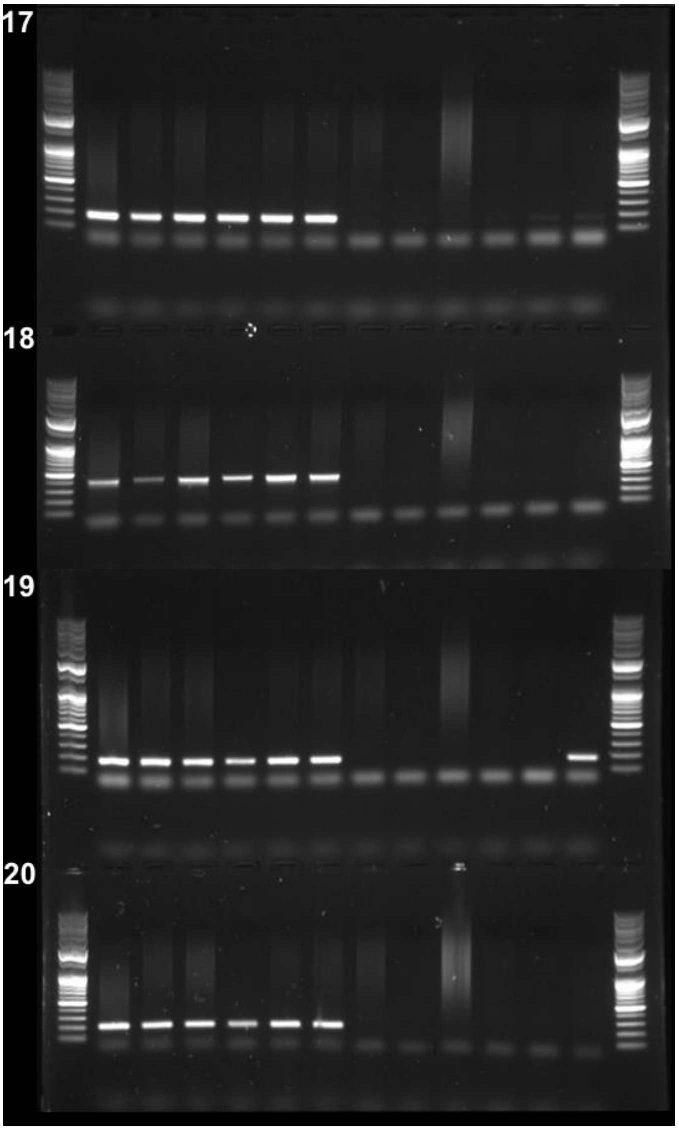

Supplementary Figure 4. PCR validation of sex-specific fragments full-length gel images. Full length sample gel electrophoresis images (from Supplementary Figure 3) of PCR for scaffolds determined to be sex-specific based on computational analyses. PCRs were tested on six females and six males, and the associated lanes are denoted with ♀ and ♂ above the first gel image, respectively. The first and last lanes are labeled with “L” to denote 100 bp ladder. Numerical labels correspond to primer information provided in Supplemental Table 3 along with the results of all PCRs tested. The following primer pairs were run on the same gel (top and bottom, respectively): 6&7, 8&9, 10&11, 12&13, 14&15, 17&18, and 19&20.

Supplementary Table 1- Coverage statistics for scaffolds that were assigned to the *Ambystoma* Z chromosome. Scaffolds identifiers correspond to the published assembly.

Supplementary Table 2- Alignment statistics and estimated coverage ratios for regions that show enrichment in females.

Supplementary Table 3- Summary of PCR assays for predicted female-specific regions.
